# Development of a new immunodiagnostic tool for poultry coccidiosis using an antigen-capture sandwich assay based on monoclonal antibodies detecting an immunodominant antigen of *Eimeria*

**DOI:** 10.1016/j.psj.2023.102790

**Published:** 2023-05-19

**Authors:** Youngsub Lee, Hyun S. Lillehoj

**Affiliations:** Animal Bioscience and Biotechnology Laboratory, Beltsville Agricultural Research Center, Agricultural Research Service, United States Department of Agriculture, Beltsville, MD 20705, USA

**Keywords:** *Eimeria*, antigen-capture ELISA, profilin, 3-1E, coccidiosis

## Abstract

This study was conducted to develop an antigen-capture ELISA that detects an immunodominant antigen of *Eimeria*, 3-1E which is present in all *Eimeria* species, using a set of 3-1E-specific mouse monoclonal antibodies (**mAbs**). Highly sensitive 3-1E-specific antigen-capture ELISA was established using compatible mAb pairs (#318 and #320) selected from 6 mAbs (#312, #317, #318, #319, #320, and #323) with high binding activity against recombinant 3-1E protein. These anti-3-1E mAbs specifically recognized *E. tenella* sporozoites and a higher level of 3-1E was detected in the lysate of sporozoites than in sporocysts. Immunofluorescence assay (**IFA**) using 2 mAbs (#318 and #320) showed specific staining around the membrane of *E. tenella* sporozoites. In order to measure the changes in the 3-1E level during in coccidiosis, serum, feces, jejunal, and cecal contents were individually collected daily for 7-days postinfection (**dpi**) with *E. maxima* and *E. tenella*. The new ELISA was sensitive and specific for 3-1E detection in all samples collected daily from *E. maxima*- and *E. tenella*-infected chickens for a week, and the detection sensitivity ranges were 2 to 5 ng/mL and 1 to 5 ng/mL in serum, 4 to 25 ng/mL and 4 to 30 ng/mL in feces, 1 to 3 ng/mL and 1 to 10 ng/mL in cecal contents, and 3 to 65 ng/mL and 4 to 22 ng/mL in jejunal contents. Following coccidiosis, the overall 3-1E levels started to increase from 4 dpi, and the highest production was shown on 5 dpi. Among the samples collected from *Eimeria*-infected chickens, the highest detection level was found in the jejunal contents of *E. maxima*-infected chickens. Furthermore, the level of IFN-γ in serum was significantly (*P* < 0.05) increased from 3 dpi and peaked on 5 dpi post *E. maxima* infection. Post *E. tenella* infection, the level of IFN-γ in serum gradually (*P* < 0.05) increased from 2 to 5 dpi and plateaued at 7 dpi. The level of TNF-α in serum was rapidly (*P* < 0.05) increased from 4 dpi and those levels were kept until 7 dpi post both *Eimeria* infections (*E. maxima* and *E. tenella*). More importantly, the daily changes in the 3-1E levels in different samples from *E. maxima-* and *E. tenella-*infected chickens were effectively monitored with this new antigen-capture ELISA. Therefore, this new immunoassay is a sensitive diagnostic tool to monitor coccidiosis in a large field population in the commercial poultry farms before clinical symptoms develop using serum, feces, and gut samples during the entire period of infection cycle starting from 1 d after infection.

## INTRODUCTION

Avian coccidiosis an intestinal disease caused by parasites of the genus *Eimeria* is an economically important disease for poultry industry worldwide (Chapman, 2014). Currently, 7 distinct species of *Eimeria* (*E. acervulina, E. brunetti, E. maxima, E. mitis, E. necatrix, E. praecox*, and *E. tenella*) which are known to infect different intestinal sites for invasion and intracellular development (Lopez-Osorio et al., 2020; Lee et al., 2022a ). Each *Eimeria* species is known to invade and propagate at different sites in the intestine, resulting in severe intestinal damage. Intestinal hemorrhage, nutrient malabsorption, diarrhea, and weight loss occur as a result of intestinal dysfunction in avian coccidiosis (Kim et al., 2019; Lillehoj and Lillehoj, 2000; Lillehoj et al., 2000). Avian coccidiosis is transmitted from one animal to another through the fecal-oral route via infectious oocysts (McMullin, 2022). In addition, mechanical carriers (e.g., clothing, equipment, insects, farm workers, and other animals) are also potential transmitting factors. The infected fecal material can contaminate eggs, feed, water, bedding material, or soil. Virtually all poultry including layer chickens and other birds reared commercially for meat production are commonly exposed to conditions conducive to developing coccidiosis (Shivaramaiah et al., 2014).

Current preventive measures for coccidiosis and other diseases associated with *Eimeria* infection such as necrotic enteritis predominantly include chemotherapy (Chapman, 2014) and conventional vaccines (Shirley et al., 2007; Shivaramaiah et al., 2014). However, due to the increasing concerns with antimicrobial resistance associated with the extensive use of antibiotics in animal agriculture, alternative and sustainable methods of coccidiosis control are timely needed. Various potential immunogenic antigens have been explored as recombinant and subunit vaccines with different degree of success to control coccidiosis experimentally (Lillehoj et al., 2000; Song et al., 2021; Lee et al., 2022a). Furthermore, many nonantibiotic alternative strategies have been developed to mitigate the negative effects of coccidiosis including natural plant-derived phytochemicals, probiotics, and hyperimmune egg yolk antibodies (Kim et al., 2013; Kim and Lillehoj, 2018; Lee et al., 2018a; Lillehoj et al., 2018; Callaway et al., 2021). However, there is a timely need for sensitive detection method for early diagnosis of coccidiosis in large poultry production system for more effective disease management to reduce economic losses associated with coccidiosis.

Up to date, numerous antigen-capture ELISA (also known as sandwich ELISA) have been developed for immunological studies in poultry, including those developed from our lab (Dai et al., 2016; Chaudhari et al., 2020a; Lee et al., 2021a; Panebra et al., 2021; Lu et al., 2022a; Goo et al., 2023). Most of these tools have focused on analyzing the immunological changes of the host against specific infection by analyzing cytokines secretion, which are signaling molecules that regulate the function of immune cells in poultry (Lu et al., 2022b). In a recent study, we have developed a new diagnostic tool to detect a major toxin protein, NetB, a toxin associated with necrotic enteritis caused by *Clostridium perfringens* type A in poultry (Lee et al., 2020a). NetB-toxin detecting sandwich ELISA can monitor NE-positive flocks in commercial poultry farms or to identify and quantify NetB protein secreted by *Clostridium* isolates in vitro (Lee et al., 2021b; Goo et al., 2023). In the current study, we focused on developing an analysis tool that can directly detect coccidiosis-infected chickens in commercial production environment using serum, gut, and fecal samples using a set of *Eimeria*-specific mAbs that recognize most immunodominant antigen of *Eimeria* parasites, 3-1E, which is present in all apicomplexan parasites (Lillehoj et al., 2000). Availability of a sensitive quantitative immunoassay that can specifically detect coccidiosis-infected chickens in large commercial poultry flocks earlier than when the diseased animals show clinical symptoms will reduce the economic cost associated with coccidiosis.

## MATERIALS AND METHODS

### Production and Characterization of mAbs Against 3-1E

The recombinant 3-1E protein expressed in *Escherichia coli* (***E. coli***) (Lillehoj et al., 2000, 2005) was used for all mice procedures including immunization and cell fusion to develop 3-1E-specific mouse monoclonal antibodies (Genscript Inc., Piscataway, NJ). The fused hybridomas producing mouse anti-3-1E mAbs were screened by indirect ELISA based on high binding affinity as previously described (Min et al., 2002). The hybridoma cell's supernatants that showed high affinity were purified by Protein G agarose chromatography (Thermo Fisher Scientific, Rockford, IL). The immunoglobulin isotyping was determined by an Iso Quick Kit (Sigma-Aldrich, St. Louis, MO) per manufacturer's instructions.

### Immunoblotting Analysis

Recombinant 3-1E (1 μg/lane) antigen was loaded onto any kD protein gels (Bio-Rad, Hercules, CA) and transferred onto nitrocellulose membranes using the Turbo Trans-Blot system (Bio-Rad, Hercules, CA). The membrane was stained with Ponceau S solution (Sigma-Aldrich) for 5 min and the visualized proteins were washed with PBS (pH 7.2) containing 0.05% Tween 20 (**PBS-T**). The membrane was blocked using SuperBlock Blocking Buffer (Thermo Scientific, Waltham, MA) for 1 h at room temperature (**R/T**) and washed 3 times with PBS-T, followed by an incubation with 1 μg/mL of mouse anti-3-1E mAbs (#312, #317, #318 #319, #320, and #323) for 2 h at R/T. Following 3 washes in PBS-T, the membranes were incubated with HRP-coupled goat anti-mouse IgG (Sigma-Aldrich) in PBS-T (1:5,000) for 1 h at 37°C, and the membranes were visualized via a ChemiDoc imaging system (Bio-Rad) using Clarity Western ECL Substrate (Bio-Rad) as a chromogenic substrate.

### Indirect ELISA

Indirect ELISA was carried out using 4 different *E. coli*-expressed recombinant proteins (Lee et al., 2020b), chIFN-γ (Yun et al., 2000), 3-1E (Lillehoj et al., 2005), chIL-10 (Lee et al., 2018b), and chTNF-α (Lu et al., 2022a). Briefly, flat-bottomed 96-well microtiter plates were coated with 1 µg/mL of each recombinant protein (3-1E, chIFN-γ, ChTNF-α, and chIL-10) overnight (**O/N**) at 4°C. The plate was washed, blocked with Superblock Blocking Buffer (Thermo Scientific, Waltham, MA) at R/T for 1 h, and incubated with 1 µg/mL of mouse anti-3-1E mAbs (#312, #317, #318 #319, #320, and #323). Following washes, the plate was incubated with HRP-conjugated goat anti-mouse IgG (Sigma-Aldrich) in PBS (1:5,000) for 1 h at 37°C. The antigen-antibody reaction was visualized by same volume of 3,3′,5,5′-tetramethylbenzidine (**TMB**) substrate (Sigma-Aldrich, St. Louis, MO) and the peroxidase reaction was stopped by 2 N H_2_SO_4_ (Sigma-Aldrich, St. Louis, MO). The absorbance (OD) was read at 450 nm by using an ELx-800 microplate reader (Biotek, Winooski, VT). All incubation steps were performed on the plate shaker, and the plates were washed 6 times with PBS-T after each step. Two independent experiments, each consisting of 3 replicates, were performed.

### Development of Antigen-Capture ELISA

Development of antigen-capture ELISA was carried out via pairing assay to select compatible mAb combinations as previously described (Lee et al., 2021a). Briefly, each mAb used for detection was labeled with biotin (EZ-Link Sulfo-NHS-BiotinTM reagent; Thermo Scientific) according to the manufacturer's instructions. The 96-well flat-bottomed plates were incubated with nonbiotinylated mAb (each 1 µg/mL) O/N at R/T. The plates were washed, blocked with Superblock Blocking buffer (Thermo Scientific, Waltham, MA) at R/T for 1 h, and incubated with 500 ng/mL of recombinant 3-1E protein for 1 h. Following washes, the plates were incubated with 1 µg/mL biotin-labeled mAbs for 1 h and conjugated with avidin-HRP (1:5,000) for 1 h after washes. The incubation steps were performed on the plate shaker and the plates were washed 6 times with PBS-T after each step. For all steps including antibody-antigen reaction, the stopping reaction and absorbance readings were carried out using the same method as the indirect ELISA. Two independent experiments, each consisting of 3 replicates, were performed. The pairing combination between the same clones (i.e., #312 as the capture antibody and Bio-312 as the detection antibody) was considered a negative control.

### Preparation of Sporocyst and Sporozoite Lysates

Freshly propagated sporulated oocysts of *E. maxima* and *E. tenella* (1 × 10^5^, respectively) were sterilized by a Clorox liquid bleach (Oakland, CA) for 30 min and washed 3 times with distilled water. Following centrifugation at 12,000 × *g* for 10 min, the sporulated-oocyst pellets were resuspended in 1 mL of PBS and the lysate of sporocysts was obtained by using a bead beater with 0.05 mm glass as described previously (Wickramasuriya et al., 2021; Lee et al., 2022a). Subsequently, the sporocysts were resuspended in the same volume (1 mL) of excystation media (0.25% trypsin, 0.014 M taurocholic acid), and the sporozoite lysate was obtained after 4-h incubation at 41°C. Both lysates (sporocyst and sporozoite) were centrifuged at 12,000 × *g* for 10 min to remove the debris and analyze the supernatants by an antigen-capture ELISA after serially 2-fold dilution in PBS or IMDM media, respectively. PBS and IMDM alone were used as the vehicle control and all values were normalized by subtracting the background color (vehicle controls). The concentration was determined by a standard curve that was established using a recombinant 3-1E protein. Two independent experiments, each consisting of 3 replicates, were performed.

### Immunofluorescence Microscopy

*E. tenella* sporozoites were fixed in suspension with 4% formaldehyde on the siliconized glass slides (Kako, Kyoto, Japan) for 30 min, followed by 3 washes in PBS. After air drying for 30 min, the specimens were permeabilized with 0.1% Triton X-100 (Sigma-Aldrich, St Louis, MO) for 10 min and incubated with blocking solution containing 3% goat serum in PBS (w/v) for 1 h at 37°C. After 3 washes, slides were incubated with either purified anti-3-1E mAbs (#318 and #320) or the positive control, 15.84 mAb (Speer et al., 1989) O/N at 4°C. Again, the specimens washed 3 times in PBS and incubated with the secondary antibody, FITC-conjugated goat anti-mouse IgG (H + L) (dilution 1:100, Jackson Immune Research) for 60 min at 37°C. Following 3 washes in PBS and 1 wash in distilled water, the slides were finally mounted with 50 µL of Fluoromount Aqueous Mounting Medium (Sigma-Aldrich). The sporozoites were visualized using Eclipse 80i fluorescence microscope (Nikon, Tokyo, Japan) with a NIS-Elements AR imaging software (Nikon).

### Animal Husbandry and Parasite Challenge Infection

All experiments were approved by the Beltsville Agricultural Research Center Institutional Animal Care and Use Committee (# 20-015). One hundred newly hatched male broiler chicks (1-day-old, Ross 708) were purchased from a local hatchery (Longnecker Hatchery, Elizabethtown, PA) and housed in Petersime brooder units maintained in a temperature-controlled closed-house environment. Feed and water were offered ad libitum during experimental periods. On d 14, chickens were weighted to adjust to the same body weight (variation within 50 g) per group and allocated to 3 groups. Each group was divided into 7 cages (7 cages per group), with 4 chickens per cage. Subsequently, chickens except in the control group were orally infected with 2 × 10^4^/bird of freshly propagated sporulated *E. maxima* or *E. tenella*, respectively. Individual body weight was recorded at d 14 before *Eimeria* challenge infection and, 4- and 7-days post *Eimeria* infection (**dpi**), and average body weight gains per group were calculated. There was no mortality during the experimental period following *Eimeria* challenge infection.

### Samples Collection and Measurement

To monitor the change of 3-1E levels, feces, serum, and jejunal and cecal contents from individual chicken were collected every 24 h for 7 d following the *Eimeria* challenge infection. Blood samples were taken in S-Monovette Gel tubes with a clotting activator (Sarstedt, Germany) by cardiac puncture. After clotting, sera were separated by centrifugation at 500 × *g* for 10 min at R/T as previously described (Lee et al., 2018a). Jejunum contents were collected from 2 equal 5 cm sections of jejunum starting at the point of Meckel's diverticulum. Digesta contents from the ceca were collected by gently squeezing each section into a tube and then pooled. The feces were collected from cloaca or the periphery of anus. For 3-1E measurements by an antigen-capture ELISA, each sample (feces, jejunal contents, cecal contents, and serum) was diluted 1:10 (100 µL/1,000 µL, v/v) in PBS and homogenized on the shaker for O/N at 4°C. Each sample was analyzed in triplicates, and the concentration was determined by a standard curve that was established using a recombinant 3-1E protein. The change of TNF-α and IFN-γ concentrations was monitored in serum samples using an ELISA analysis with its specific mAbs which were previously established by our lab (Yun et al., 2000; Lu et al., 2022a ).

### Statistical Analysis

Statistical analysis was performed using SPSS 20.0 statistical software (SPSS Inc., Chicago, IL) for Windows. All data, except body weight, were evaluated using Student *t* test, and the body weight difference between groups was evaluated using 1-way ANOVA followed by Tukey's test. *P* < 0.05 was considered a significant difference.

## RESULTS

### Production of Mouse Anti-3-1E mAbs

**Figure 1:**
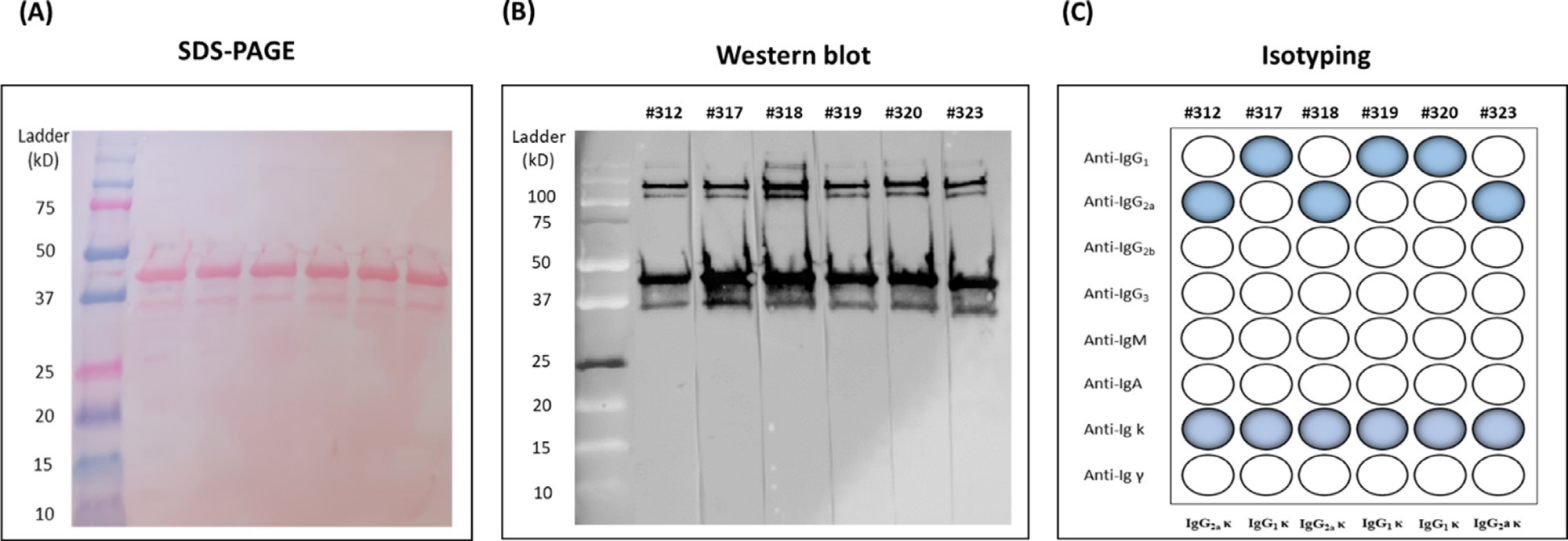
Verification of purified mouse anti-3-1E mAbs against recombinant 3-1E. (A) SDS-PAGE of recombinant *E. coli*-expressed 3-1E showed a specific band between 37 and 50 kDa. Molecular weight (kDa) marker is presented on the left. (B) Anti-3-1E monoclonal antibodies (#312, #317, #318, #319, #320, and #323) specifically detected the 3-1E band. (C) Isotype analysis indicated that 3 mAbs (#317, #319, and #320) were of the IgG_1_Ƙ, and another 3 mAbs (#312, #318, and #323) were of the IgG_2a_Ƙ.

Six hybridomas (#312, #317, #318 #319, #320, and #323) showing high binding activities to 3-1E protein were cloned and selected for further study. The Indirect ELISA results showed that all anti-3-1E mAbs that we tested in this study showed strong affinity to recombinant 3-1E protein without cross-reaction with other control recombinant proteins (Table 1). The specificity of all 6 mAbs to 3-1E protein was confirmed by Western blot (Figure 1A and B), and the isotyping results indicated that 3 mAbs (#317, #319, and #320) were of the IgG_1_Ƙ and another 3 mAbs (#312, #318, and #323) were of the IgG_2a_Ƙ (Figure 1C).

**Table 1:** Characterization of monoclonal antibodies specificity to recombinant 3-1E.

	Reactivity of chicken cytokines OD 450 (nm)^1^			
mAb	3-1E	chIFN-γ	chTNF-α	chIL-10
#312	1.209 ± 0.053	0.088 ± 0.006	0.062 ± 0.005	0.058 ± 0.007
#317	1.333 ± 0.055	0.077 ± 0.003	0.050 ± 0.007	0.056 ± 0.004
#318	1.454 ± 0.034	0.067 ± 0.006	0.050 ± 0.008	0.056 ± 0.008
#319	0.947 ± 0.050	0.059 ± 0.007	0.052 ± 0.009	0.059 ± 0.012
#320	1.503 ± 0.017	0.050 ± 0.008	0.070 ± 0.003	0.071 ± 0.002
#323	1.136 ± 0.028	0.052 ± 0.005	0.071 ± 0.004	0.079 ± 0.004

1 *E. coli*-expressed recombinant 3-1E, chIFN-γ, chTNF-α, and chIL-10 were used as the coating antigen (1.0 µg/mL) in the indirect ELISA analysis. Each indicated mAbs (1.0 µg/mL) was incubated with coated antigens, and their binding was detected with HRP-labeled goat anti-mouse IgG. Two independent experiments, each consisting of 3 replicates, were performed. Values are means ± SD.

### Establishment of Antigen-Capture ELISA

**Figure 2:**
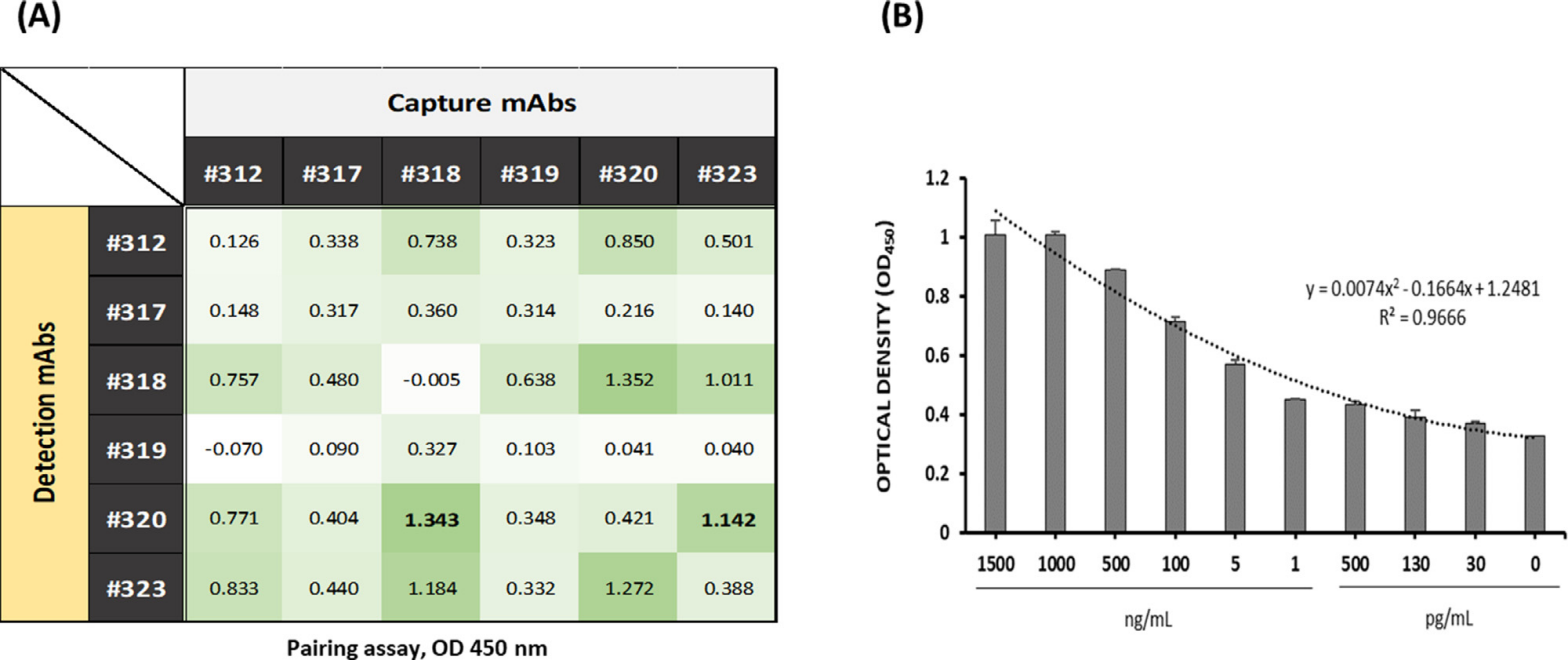
Evaluation of the 3-1E mAbs by pairing assay and an established standard curve. (A) Six monoclonal antibodies were biotinylated and used as detection antibodies to determine the best antibody pairing combination for sandwich ELISA development. The plated was coated with same concentration of each mAb (1 µg/mL) as a capture antibody followed by incubation with 500 ng/mL of recombinant 3-1E protein. Then, the same concentration of each biotinylated antibody (1 µg/mL) was added as the detection antibody and the signal was amplified by treatment of avidin-HRP. The paring combination between same clones (i.e., #312 as the capture antibody and Bio-312 as the detection antibody) was considered as negative controls. Based on the results in the pairing assay, 2 combinations (#318/Bio-320 or #323/Bio-320) were selected. (B) Standard curve was established using a #318/Bio-320 combination and the detectable range also was verified. Error bars indicate mean ± SD.

Two different antigen-capture ELISAs detecting recombinant 3-1E protein were developed (Figure 2A). Two mAb combinations showing strong affinity were found in the pairing assay with 2 mAbs (#318 or #323) as a capture Ab and biotinylated #320 mAb as a detection Ab. The standard curve was established using a combination of #318/biotinylated #320, allowing detectable ranges to have a maximum 1 µg/mL and minimum 30 pg/mL (Figure 2B). A combination of #318/biotinylated #320 showed a lower background with slightly stronger affinity to recombinant 3-1E protein compared to that of #323/biotinylated #320 combination as capture and detection antibodies, respectively. Hence, a combination of #318/biotinylated #320 was used in our new ELISA to measure 3-1E in the clinical samples which were collected from *Eimeria*-infected chickens. Similar results were obtained when ELISA was repeated using the mAb combination of #323/biotinylated #320 as capture and detecting antibodies, respectively.

### Measurement of 3-1E at Different Development Stages of *Eimeria* by Antigen-Capture ELISA

**Figure 3:**
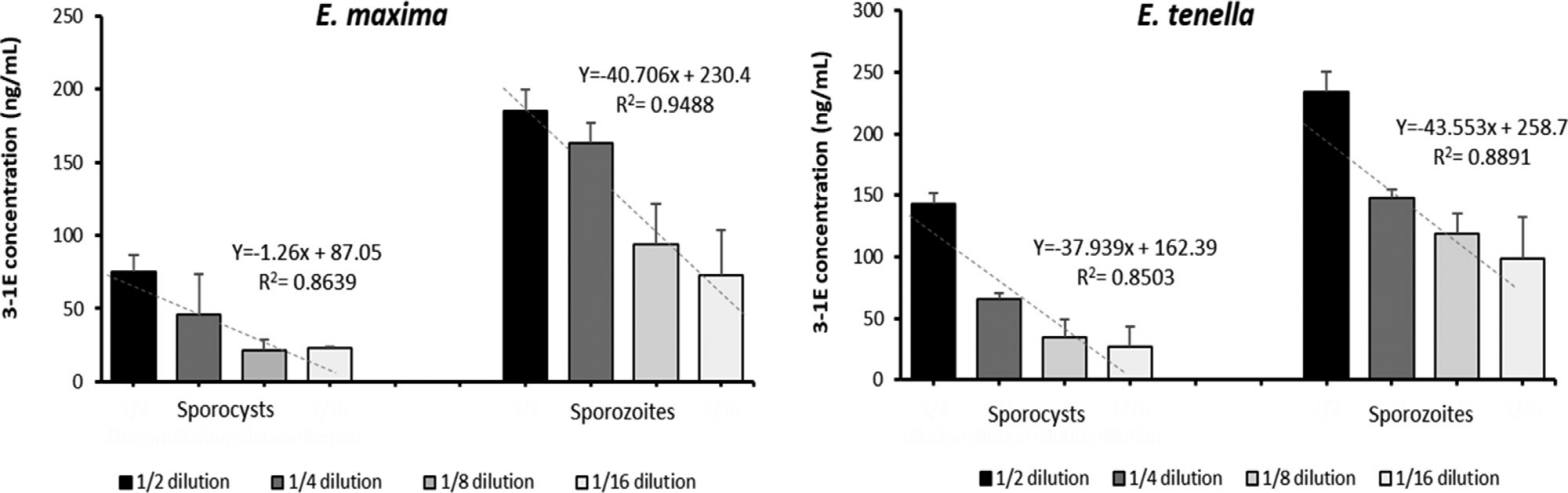
Sandwich ELISA to detect 3-1E at different development stages of *E. maxima* and *E. tenella*. The lysate of sporocysts was obtained from freshly propagated sporulated oocysts (*E. maxima* and *E. tenella*), respectively, by a bead beating method. The lysate of sporozoites was obtained from the sporozoites released in excystation media that were cultured with the same volume of sporocysts isolated from *E. maxima* and *E. tenella*. The lysates of sporocysts and sporozoites were serially diluted in PBS or IMDM-based excitation media, respectively. The level of 3-1E was analyzed using a sandwich ELISA (#318/Bio-320). PBS and IMDM alone were used as the vehicle control. All values were normalized by subtracting the background color (vehicle controls). The concentration was determined by a standard curve that was established using a recombinant 3-1E protein. Two individual experiments were performed, and data were analyzed in triplicate. Each bar represents the mean ± SD of triplicate samples.

The level of 3-1E was measured in the lysates of sporocysts and sporozoites of the sporulated-oocysts of *E. maxima* and *E. tenella* by a combination of #318/biotinylated #320. As shown in Figure 3, the 3-1E antigen was detected in both lysates (sporocyst and sporozoites) in a dose-dependent manner. The detected level of 3-1E was higher in the lysates of sporozoites than of sporocysts in both *E. maxima* and *E. tenella* with *E. tenella* showing generally higher binding than *E. maxima*.

### Immunofluorescence Microscopy Assay

**Figure 4:**
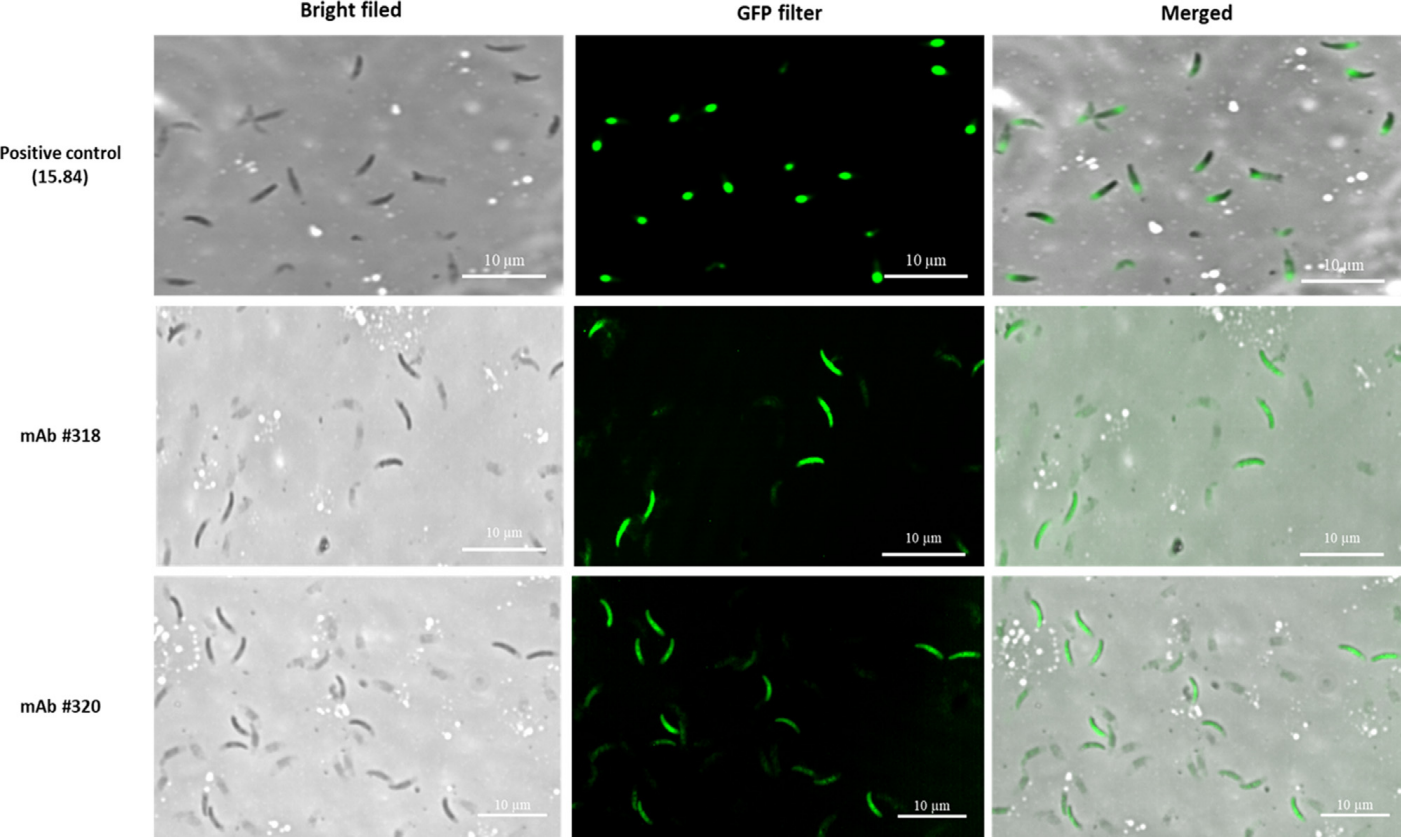
Localization of 3-1E on sporozoites using anti-3-1E mAb #318 and #320 by IFA. *E. tenella* sporozoites were incubated with anti-3-1E mAbs (#318, #320), or 15.84 mAb (positive control). FITC-conjugated goat anti-mouse IgG (H + L) was used to visualize FITC-stained parasites under immunofluorescence microscopy. Horizontal bar represents 10 µm.

Using both anti-3-1E #318 and #320 mAb as the first antibody and 15.84 mAb as a positive control, the localization of 3-1E in sporozoites was studied by an immunofluorescence assay as previously described (Sasai et al., 2008). As presented in Figure 4, the results showed that 3-1E in *E. tenella* sporozoites were recognized by anti-3-1E #318 and #320 mAbs mainly on the surface of sporozoites. The positive control (15.84 mAb) also recognized the *E. tenella* sporozoite, however, unlike the recognition site of anti-3-1E #318 and #320 mAbs, only one end of *E. tenella* sporozoite was stained in a round shape. A merged picture of both modes (bright filed and GFP filter) accurately indicated the regions recognized by anti-3-1E mAbs (318 and #320) and positive control, respectively.

### Body Weight Change of Chickens Postchallenge Infection With *E. Maxima* and *E. Tenella*

Body weight changes after *E. maxima* and *E. tenella* challenge infections are presented in Table 2. The initial body weight had no significant difference and the standard deviation between groups was within the 20 g. *E. maxima* challenge infection significantly (*P* < 0.05) reduced body weights at 4 dpi, but there was no significant difference at 7 dpi. *E. tenella* challenge infection significantly (*P* < 0.05) reduced body weights at 4 dpi and 7 dpi compared to unchallenged chickens.

**Table 2:** Body weight (g) change of chickens following *E. maxima* and *E. tenella* challenge infections.

Days	Control	*E. maxima*	*E. tenella*	SD (±)	*P* value
D 14 (initial)	701.2	696.4	698.9	18.63	0.603
D 18 (4 dpi)	1033.9^a^	974.0^b^	957.8^b^	52.54	<0.01
D 21 (7 dpi)	1217.5^a^	1147.2^ab^	1099.8^b^	70.79	0.041

Abbreviations: D, day; dpi, days post-infection; SD, standard deviation.

All chickens except the control group were infected by oral gavage at day 14 with 20,000 oocysts/chicken of *E. maxima* and *E. tenella*. Individual body weight change was recorded at d 14, d 18, and d 21.

a,b Means in the same row with different superscripts differ (*P* < 0.05) and the difference was evaluated using one-way ANOVA followed by Tukey's test.

### 3-1E Level Change in Chickens Postchallenge Infection With *E. Maxima* and *E. Tenella*

**Figure 5:**
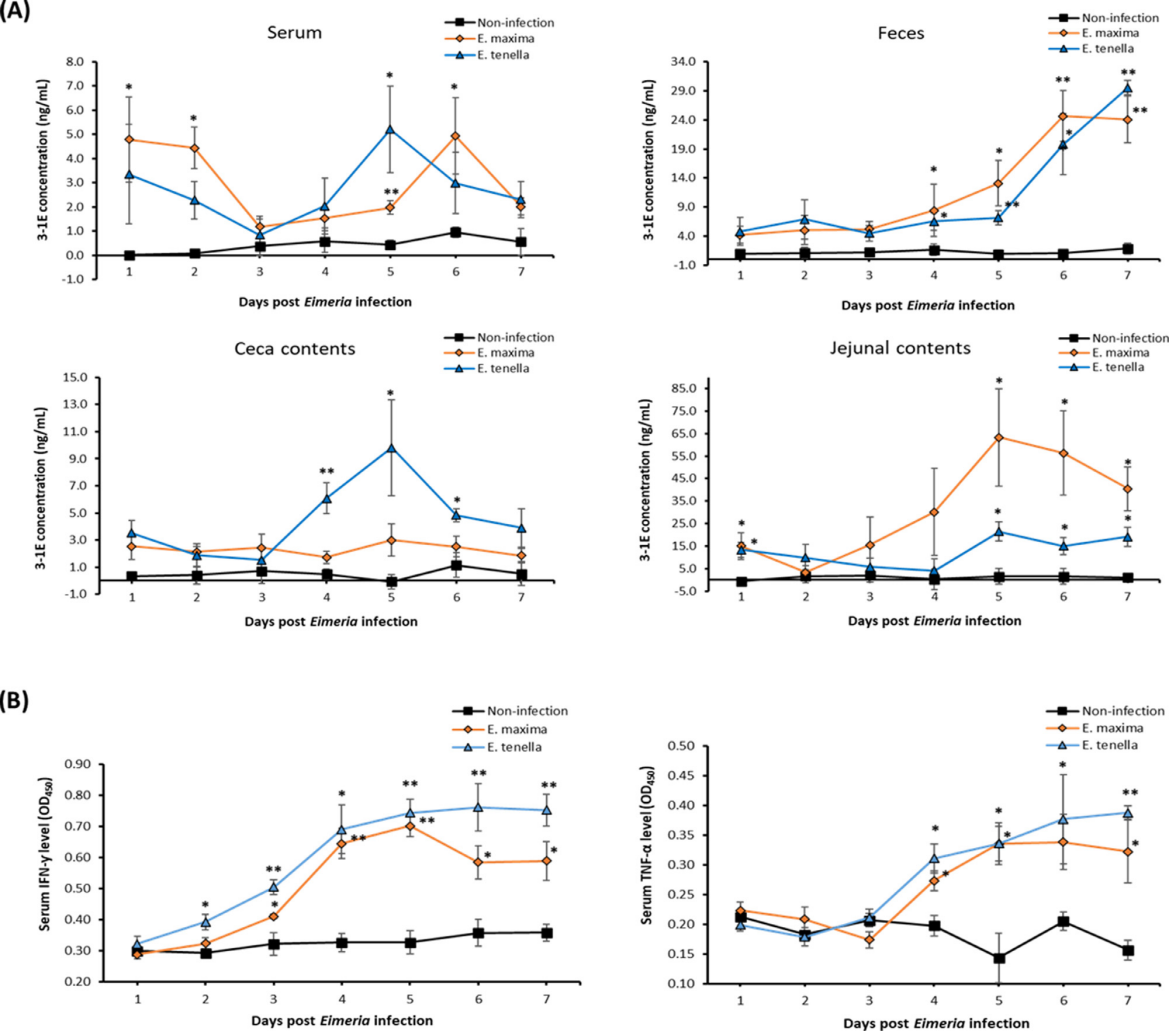
(A) Detection of 3-1E levels in serum, feces, ceca, and gut digesta of *E. maxima*- and *E. tenella*-challenged chickens. On d 14, chickens were orally challenged with *E. maxima* and *E. tenella*, and blood, feces, ceca, and gut digesta samples were collected every 24 h for 7 d. The daily changes of 3-1E level in each sample (*n* = 4/group) were analyzed by our new sandwich ELISA (318/Bio-320). Then, the concentration was determined by a standard curve that was established using recombinant 3-1E protein. (B) The daily pattern change of serum TNF-α and IFN-γ levels. On d 14, chickens were orally challenged with *E. maxima* and *E. tenella*, and blood samples were collected every 24 h for 7 d. The daily pattern changes of TNF-α and IFN-γ in serum (*n* = 4/group) were analyzed by a sandwich ELISA for TNF-α and IFN-γ, respectively. Each bar represents the mean ± SEM of duplicate samples, *P < 0.05 and **P < 0.01 represent significant differences compared to nonchallenged chickens, respectively.

The 3-1E levels measured by a combination of #318/biotinylated #320 in serum, feces, together with cecal and jejunal contents from chickens post-*E. maxima* and *E. tenella* challenge infection are shown in Figure 5A. The 3-1E level of uninfected chickens was kept at between 0 and 1 ng/mL without any significant change in all samples (serum, feces, cecal, and jejunal contents). Overall, in the samples collected from *Eimeria*-infected chickens, the jejunal contents infected with *E. maxima* showed highest detection levels.

In serum, the level of 3-1E was significantly (*P* < 0.05) increased on d 1, 2, 5, and 6 post-*E. maxima* infection. A similar pattern was observed in serum postinfection with *E. tenella*, but a statistical difference (*P* < 0.05) was found only at 5 dpi compared to uninfected chickens. In feces, the level of 3-1E was significantly (*P* < 0.05) increased from d 4 to 6 and stayed at similar levels from d 6 post-*E. maxima* infection. Postinfection with *E. tenella*, the daily 3-1E level changes in feces showed an analogous pattern to those of chickens infected with *E. maxima* except that the 3-1E level further increased to 7 dpi than 6 dpi. Postinfection with *E. tenella*, the level of 3-1E in cecal contents significantly (*P* < 0.05) increased from d 4 to 5 and rapidly decreased from d 6. In contrast with chickens infected with *E. tenella*, there was no significant difference on daily pattern postinfection with *E. maxima*. In jejunal digesta, the level of 3-1E was significantly (*P* < 0.05) increased on d 1, 5, 6, and 7 post-*E. maxima* infection. The level of 3-1E peaked at 5 dpi and then gradually decreased. The level of 3-1E in jejunal digesta post-*E. tenella* infection showed a similar pattern to chickens infected with *E. maxima*. However, on average, the detected 3-1E amount was much lower than those of chickens infected with *E. maxima*.

### IFN-γ and TNF-α Level Change in Chickens Postinfection With *E. Maxima* and *E. Tenella*

IFN-γ and TNF-α levels measured in the serum of chickens’ post-*E. maxima* and *E. tenella* challenge infections are shown in Figure 5B. Postinfection with *E. maxima*, the level of IFN-γ in serum significantly (*P* < 0.05) increased from 2 to 7 dpi. Postinfection with *E. tenella*, the serum IFN-γ level was rapidly increased (*P* < 0.05) from 3 dpi to 5 dpi and gradually decreased from 6 dpi. The graph of TNF-α level change in serum showed a similar pattern to that of IFN-γ. However, statistical significance (*P* < 0.05) on the TNF-α increase was found starting from d 4 postinfection with *E. maxima* and *E. tenella*.

## DISCUSSION

In the current study, 6 new mouse mAbs that specifically identify an immunodominant protein of *Eimeria* parasites, 3-1E, which is a cross-protective antigen of *Eimeria* spp., to develop an antigen-capture ELISA to detect coccidiosis-infected chickens. All purified anti-3-1E mAbs that we initially selected specifically recognized a 3-1E recombinant protein without any cross-reaction to unrelated control antigens by indirect ELISA (Table 1) and Western blot analysis (Figure 1A and B). In addition, the immunofluorescence microscopy demonstrated that #318 and #320 mAbs which were used in the new antigen-capture ELISA, identified a surface antigen of *E. tenella* sporozoites (Figure 4.). More importantly, this new immunoassay can effectively monitor the daily changes in the 3-1E antigen level in various clinical samples collected from *Eimeria*-infected chickens postchallenge infection with *E. maxima* and *E. tenella*.

Overall, the mAb #320 showed the highest specific binding activity with 3-1E recombinant protein among the 6 mAbs that we selected and thus used as the detecting mAb for antigen-capture ELISA (Figure 2). These antibodies showed surface staining on the sporozoite and sporocyst stages of *Eimeria* parasites and reacted to a 19 kDa protein in the lysates of sporocysts and sporozoites (data not shown). As shown in our previous studies (Lee et al., 2020b, 2021a), the combination of 2 different antibodies with specificity to the same antigen makes a good antibody pair for sandwich antigen-capture immunoassay (Figure 1C). A combination of #318 mAb as the antigen-capture antibody and the biotinylated #320 mAb as the detecting antibody made 3-1E-specific ELISA with high sensitivity with a lower background. This new ELISA was used to detect the daily changes in the 3-1E levels in feces, serum, and gut contents in broiler chickens post-*E. tenella* infection. Similar results were seen in a repeating analysis by a combination of #323/biotinylated #320 mAbs in serum from *E. maxima*-infected chickens (data not shown). Postinfection with *E. maxima*, a rapid increase of 3-1E level was detected starting from d 4 in the feces and intestinal contents (Figure 5A). However, in serum samples from *E. maxima-*infected chickens, significantly (*P* < 0.05) increased levels of 3-1E were detected on 1 and 2 dpi with *Eimeria* infection. The different kinetics of 3-1E detection in different samples may be due to the release of circulating soluble parasite proteins after coccidiosis infection.

Since *E. maxima* parasites undergo its life cycle in the jejunum, the level of 3-1E in the cecal contents collected from *E. maxima*-infected chickens was much lower compared to that of *E. tenella*-infected chickens (Dubey and Jenkins 2018; Park et al., 2020; Lee et al., 2022b). Although there was no significant difference, coccidiosis-infected chickens showed a higher 3-1E level than the uninfected chickens. Overall, in postinfection with *E. tenella*, the daily 3-1E level changes showed a similar pattern to that of chickens infected with *E. maxima* in serum, feces, and jejunal digesta. Following *E. tenella* infection, chickens showed the highest 3-1E level in the cecal content on d 5 postinfection when the intestinal lesion was most severe and a gradual decrease from d 6 postinfection. Higher level of 3-E antigen in the ceca following *E. tenella* infection is compatible with the established notion that the ceca is the primary site of *E. tenella* replication (Macdonald et al., 2017; Choi et al., 2021; Lee et al., 2022b). However, since 3-1E antigen expression was seen mostly associated with the early stages of coccidia life cycle, especially sporozoites and sporocysts, the decreased 3-1E levels seen from d 4 postinfection with *E. tenella* may reflect a reduced expression of 3-1E in later stage of oocyst life cycle. Furthermore, 3-1E is an immunodominant antigen whose expression initiates an early stage of host inflammatory response that leads to protective cell-mediated immune response (Lillehoj et al., 2005). Unlike previous infections where we used 1 × 10^4^ oocysts for infection (Chaudhari et al., 2020b; Lu et al., 2022a), in the present study, a higher dose (2 × 10^4^ oocyst per chicken) was used to monitor a daily 3-1E level changes by an antigen-capture ELISA post-*Eimeria* infection. Based on body weight changes (Table 2), the chickens infected with *E. tenella* did not recover on 7 dpi compared to uninfected chickens. Subsequently, we measured the daily change of IFN-γ and TNF-α levels in serum to monitor the kinetic response of systemic inflammatory reaction following *Eimeria* challenge infection (Figure 5B) since 3-1E is an immunodominant antigen of *Eimeria* inducing early host inflammatory response (Lillehoj et al., 2000, 2005). From d 2 or 3 postinfection with *E. tenella*, the level of IFN-γ and TNF-α gradually increased and sustained its elevated level until 7 dpi. These results suggest that from d 2 of *E. tenella* infection, there was significant damage to the intestinal epithelial cells and systematic inflammatory responses. Similar to this study, it was reported that the levels of IFN-γ and TNF-α in serum gradually elevated from d 3 post-*Eimeria* infection (*E. maxima* and *E. tenella*) and those levels were kept high for 10-days postinfection (Yun et al., 2000; Hong et al., 2006a,b; Lu et al., 2022a). Among all samples, the most similar graph patterns between *E. maxima* and *E. tenella* infections were found in fecal samples. In both *Eimeria* infections, the 3-1E level increased from d 5 reaching the highest level on d 6 or 7 postinfection. Interestingly, this graph pattern in the level of 3-1E is similar to a previous study monitoring a daily fecal oocyst shedding pattern post *E. maxima* and *E. tenella* infections (Cha et al., 2018; Schneiders et al., 2019).

3-1E, also known as profilin, is a soluble cytoplasmic protein widely distributed in apicomplexan parasites in both animals and plants, regulates the polymerization of actin filaments (Schluter et al., 1997). Importantly, profilins are an essential component in apicomplexan parasites that facilitates parasite invasion of host cells (Plattner et al., 2008) and a critical antigen that initiates host immune response (Lee et al., 2012). The profilin from *E. tenella* was found to be synthesized in all stages of *E. tenella* life cycle (Ding et al., 2004; Jang et al., 2011), and has been considered as a potential vaccine candidate for controlling avian coccidiosis because of their capacity to polymerize actin for host invasion by *Eimeria* parasites (Lee et al., 2012; Jang et al., 2013; Lillehoj et al., 2017). Moreover, *E. acervulina* profilin induced high levels of IFN-γ production by splenic T cells suggesting this antigen a suitable vaccine candidate for coccidiosis in poultry (Lillehoj et al., 2000, 2005; Hedhli et al., 2009). The profilin from *Toxoplasma gondii* (***T. gondii***) that formulated with oligomannose-coated liposomes reduced the parasite burden in the *T. gondii*-infected mice brain and increased the survival of the mice by enhancing IFN-γ production and IgG antibodies (Tanaka et al., 2014). IFN-γ is considered as a key moderator of cell-mediated immunity and mainly involves in host proinflammatory response (Otani et al., 2021). Therefore, we monitored the diurnal changes of serum IFN-γ levels in *Eimeria*-infected chickens in order to understand the host-parasite interaction in coccidiosis (Figure 5B). Increased levels of IFN-γ were observed from 2 dpi and 3 dpi in *E. tenella* and *E. maxima* infection, respectively. Interestingly, identical to the IFN-γ, 3-1E levels changed in the cecal contents in *E. tenella*-infected and jejunal contents in *E. maxima*-infected chickens (Figure 5A). Moreover, TNF-α, a proinflammatory cytokine that is responsible for a signaling events within cells leading to necrosis or apoptosis (Lu et al., 2022a), was increased from 4 dpi onward in both *Eimeria*-infected chickens. These time kinetic response of proinflammatory cytokine precedes the event of protective cell-mediated immunity in *Eimeria*-infected chickens (Figure 5B).

IFA was performed with indirect method using 2 mAbs detecting 3-1E (#318 and #320) and 15.84 mAb (ATCC No. HB8335) which detects a surface *Eimeria* protein with a FITC-conjugated goat anti-mouse IgG (H + L). Immunofluorescent localization by both #318 and #320 mABs showed that 3-1E was located on the surface of the sporozoite whereas 15.84 mAb stained a small region of the apical tip of *E. tenella* sporozoites, which is a same recognition site as previously described by Speer et al. (1989) and the mAb 8C-3 described by Constantinoiu et al. (2003). Although the IFA was not carried out with *E. maxima* sporozoites, it is expected that similar results will be obtained since 3-1E is a surface antigen which is found on both merozoites and sporozoites of *E. acervulina* and *E. maxima* (Venkatas and Adeleke, 2019; Lee et al., 2022b). Combination of #318 and biotinylated #320 mAbs detected 3-1E antigen in both lysates of sporozoite and sporocyst obtained from *E. maxima* (Figure 3). Although we did not test merozoites in this study, we believe that the 3-1E would also be detected from lysate of merozoites since 3-1E identified as a surface protein expressed in both stages of sporozoite and merozoite (Song et al., 2000; Tang et al., 2018).

## CONCLUSIONS

In summary, our results showed that 3-1E antigen-specific ELISA based on 2 mAbs, #318 and #320, can be effectively used to monitor the host disease response in coccidiosis and in other apicomplexan infections since profilin is an immunodominant antigen present in all apicomplexan parasites. Further validation of this immunoassay in large field trials will be required to better utilize this immunoassay for field monitoring of coccidiosis outbreak in poultry and livestock.
